# Environmentally Friendly and Multifunctional Shaddock Peel-Based Carbon Aerogel for Thermal-Insulation and Microwave Absorption

**DOI:** 10.1007/s40820-021-00635-1

**Published:** 2021-04-05

**Authors:** Weihua Gu, Jiaqi Sheng, Qianqian Huang, Gehuan Wang, Jiabin Chen, Guangbin Ji

**Affiliations:** 1 College of Materials Science and Technology, Nanjing University of Aeronautics and Astronautics, Nanjing, 210016 People’s Republic of China; 2 Shenyang Aircraft Design Institute Yangzhou Collaborative Innovation Research Institute Co., Ltd, Shenyang, 225002 People’s Republic of China

**Keywords:** Microwave absorption, Thermal insulation, Carbon aerogel, Radar cross-sectional simulation, Multi-function

## Abstract

**Highlights:**

- The eco-friendly shaddock peel-derived carbon aerogels were prepared by a freeze-drying method.
- Multiple functions such as thermal insulation, compression resistance and microwave absorption can be integrated into one material-carbon aerogel.
- Novel computer simulation technology strategy was selected to simulate significant radar cross-sectional reduction values under real far field condition..

**Abstract:**

Eco-friendly electromagnetic wave absorbing materials with excellent thermal infrared stealth property, heat-insulating ability and compression resistance are highly attractive in practical applications. Meeting the aforesaid requirements simultaneously is a formidable challenge. Herein, ultra-light carbon aerogels were fabricated via fresh shaddock peel by facile freeze-drying method and calcination process, forming porous network architecture. With the heating platform temperature of 70 °C, the upper surface temperatures of the as-prepared carbon aerogel present a slow upward trend. The color of the sample surface in thermal infrared images is similar to that of the surroundings. With the maximum compressive stress of 2.435 kPa, the carbon aerogels can provide favorable endurance. The shaddock peel-based carbon aerogels possess the minimum reflection loss value (*RL*_min_) of − 29.50 dB in X band. Meanwhile, the effective absorption bandwidth covers 5.80 GHz at a relatively thin thickness of only 1.7 mm. With the detection theta of 0°, the maximum radar cross-sectional (RCS) reduction values of 16.28 dB m^2^ can be achieved. Theoretical simulations of RCS have aroused extensive interest owing to their ingenious design and time-saving feature. This work paves the way for preparing multi-functional microwave absorbers derived from biomass raw materials under the guidance of RCS simulations.
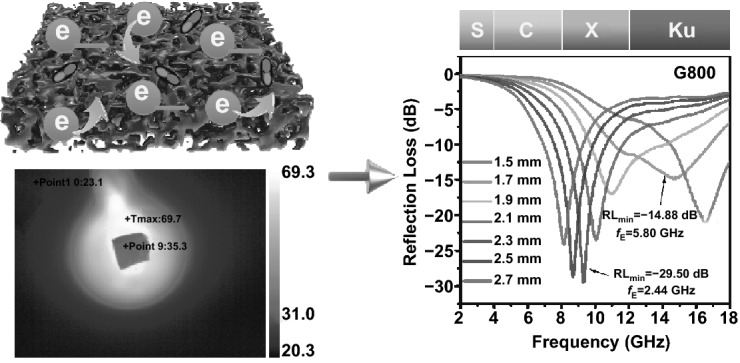

## Introduction

Recently, various materials to solve the problem of severe electromagnetic pollution have been widely researched in depth [1, 2]. Generally speaking, these materials can be broadly divided into two categories: magnetic absorbing materials and dielectric absorbers. Due to unsatisfied high density, magnetic absorbers cannot meet lightweight requirements [3, 4]. Besides, permeability usually decreases a lot with the increase of frequency because of Snoek limit, which impairs microwave attenuation ability [5, 6]. Thus, dielectric absorbing materials are ideal choices for the purpose of light weight.

Despite a variety of dielectric microwave absorbing materials and electromagnetic interference (EMI) shielding materials such as graphene [7, 8], MXene [9, 10], and carbon nanotubes [11, 12] have been investigated by academicians. However, most of the works merely focus on expanding bandwidth and improving reflection loss intensity, instead of quickly adapting complex practical environment and preliminarily designing nano/micro/macro-structure as well as predictably simulating radar cross section (RCS) [13]. For instance, Cu nanowire@graphene core–shell aerogels synthesized by Wu et al. exhibit enhanced mechanical property, including robustness, strength and modulus [14]. This kind of stable material with three-dimensional (3D) network structures has outstanding durability, which can be potential applicants for industrial manufacture. Liu et al. prepared MXene/polyimide aerogels with splendid thermal insulation and resistance performance, which can be used as ideal candidates in high-temperature working environment [15]. In addition, our group previously fabricated three-dimensional ZIF-67-coated melamine foams with excellent thermal infrared stealth property, effectively preventing targets from being detected [16]. More importantly, Chen and his co-workers synthesized mesoporous carbon fibers and used computer simulation technology (CST) to simulate important radar cross-sectional (RCS) reduction data, this greatly contributes to the pre-design of macrostructure and pre-choice of materials [17]. Therefore, multifunctional applications and elaborate design as well as numerical simulation have promising prospects for the microwave absorbing materials.

Unsatisfactorily, poisonous, harmful reagents and ingredients are often utilized to prepare microwave absorbers and EMI shielding materials [18]. Thanks to low toxicity and adequate sources, green biomass materials and their derivatives have attracted extensive attention of the researchers. For example, Wang et al. fabricated annealed sugarcane/rGO hybrid foams with superior EMI shielding effectiveness of 53 dB [19]. Qiu and his co-workers successfully prepared walnut shell-derived porous carbon microwave absorbing materials with a strong intensity of -42.4 dB at the matching thickness of 2 mm [20]. Interestingly, Dong et al. synthesized wood-based microwaving absorbers with the optimum absorption frequency range of 5.26 GHz [21]. Besides, our group also carried out deepgoing study about biomass-based microwave absorbing materials like wheat flour [22] and cotton [23]. Taking environment protection and sustainable development into consideration, we employed eco-friendly shaddock peel as origin of carbon in this work. Since shaddock peel is renewable and low-cost, this kind of biological waste can be made full use of. Benefiting from the three-dimensional porous network structure and dielectric component, fresh shaddock peel derived conductive carbon aerogels inherit unexceptional advantages, including efficient dielectric loss capacity, superb light weight characteristic and superior thermal insulation property [24]. In addition, high resistance to compression is in favor of reusing, which make biomass-based carbon aerogels can afford the high requirement of ideal microwave absorbers.

In this work, we reported shaddock peel-derived carbon aerogel via freeze-drying method and subsequent annealing procedure. As a rule, facile freeze-drying method guarantees the integral 3D skeleton architecture with excellent electrical conductivity, contributing to strong dielectric loss ability [25]. On one hand, abundant holes left by sublimation of water in raw shaddock peel can enhance the number of dipoles as well as dipole polarization, on the other hand, the high porosity can give rise to light feature in weight [26]. Therefore, the as-synthesized sample achieves the effective bandwidth of 5.80 GHz at only 1.7 mm. Furthermore, multiple functions, such as outstanding thermal stealth, heat insulation and compression resistance, make it possible for microwave absorbing materials to be efficiently applied in a variety of complex situation. Similar colors can be seen from the sample surface and the surroundings in thermal infrared images, indicating excellent thermal stealth property of the carbon aerogel. When setting 70 °C of the heating platform, the surface temperatures of the biomass-based carbon aerogel show a slow upward trend of 31.8, 33.7, and 35.3 °C within 3 min, which can be attributed to effective thermal insulation performance. With regard to mechanical property, the shaddock peel-based aerogels can provide the maximum compressive stress of 2.435 kPa at a strain of 80%. In addition, the optimum RCS reduction values of the carbon aerogel can be obtained as 16.28 dB m^2^ when setting the detection theta as 0°. Last but not least, CST simulation strategy can not only give design train of thought of microwave absorbers, but also save the experiment time of actual operation.

## Experimental

### Preparation of Shaddock Peel-Based Aerogel

**Fig. 1:**
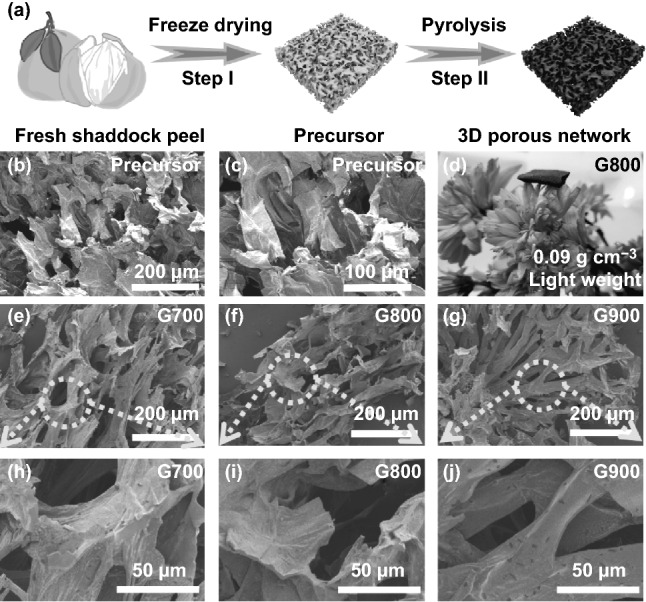
**a** Schematic of the formation process of shaddock peel-based aerogel. **b**, **c** SEM images of the 3D lightweight precursor. **d** Digital photograph of G800 sample on petals. **e**–**j** SEM images of G700, G800, and G900

Fresh shaddock purchased from school fruit store. Firstly, the entire grapefruit outer skin was peeled off and cleaned by a certain amount of deionized water and then gently wiped with non-crumb paper towels. Figure 1a exhibits the facile experiment process, which can be summarized in two steps. Step I: the prepared pomelo peel was immediately put into freeze-drying machine with a pre-freezing process for 6 h and a subsequent drying procedure for 48 h. Step II: the as-made grapefruit precursor was transferred into corundum porcelain boat and then annealed at 700, 800, and 900 °C for 2 h under Ar atmosphere with a heating rate of 2 °C min^−1^, which can be labeled as G700, G800, and G900, respectively.

### Materials Characterization

The microstructures of the samples were achieved by field emission scanning electron microscopy (FE-SEM, Hitachi S4800). The composition and phase of the 3D porous specimens were carried out by a Bruker D8 ADVANCE X-ray diffractometer (XRD) equipped with Cu K*α* radiation (*λ* = 1.5604 Å). A confocal Renishaw inVia Raman microscope was used to characterize the degree of carbonization of the as-prepared 3D porous aerogels. Electrochemical impedance spectroscopy (EIS) test of the samples was implemented by a CHI 660D electrochemical workstation to investigate the electron transport characteristics. Self-assembly tesla wireless transmission device was adopted to indirectly observe the electrical conductivity of the shaddock peel-derived carbon aerogels. The information of the pore volume, porosity and pore diameter of the samples can be obtained via mercury intrusion method using a MicroActive AutoPore V 9600 2.03.00 machine. The digital images of thermal insulation performance of the lightweight biomass-based aerogels were photographed by a thermal infrared imaging device (TVS-2000MK). The compression resistance of the shaddock peel-based 3D porous aerogel (2 × 2 × 0.5 cm^3^) was tested by an electronic universal testing machine (CMT5105, XinSanSi Enterprise Development Co. Ltd., China). During the frequency range of 2–18 GHz, electromagnetic parameters of complex permeability and permittivity were recorded by an Agilent PNA N5244A vector network analyzer using coaxial-line method at room temperature. In this test, the toroidal rings were prepared by mixing 20 wt% intact shaddocks peel-based aerogel with 80 wt % paraffin in a precise mold (*φ*_out_: 7.00 mm, *φ*_in_: 3.04 mm).

### RCS Simulation

Take actual far-field response of the shaddock peel-based aerogel materials into consideration, CST Studio Suite 2019 was used for simulating the RCS of the microwave absorber. According to widely accepted metal back model, the simulation model of the specimens was established as a square (20 × 20 cm^2^) with dual layers. In detail, the upper set as 1.7 mm signifies the absorbing layer and the bottom set as 1.0 mm is the perfect conductive layer (PEC). The shaddock peel-based aerogel-PEC model plate is placed on the *X*-O-*Y* plane, and linear polarized plane electromagnetic waves incident from the positive direction of the *Z* axis to the negative direction of the *Z*-axis. Meanwhile, the direction of electric polarization propagation is along the *X*-axis. With the open boundary conditions setting in all directions, the chosen field monitor frequency was 12 GHz. It is generally accepted that the scattering directions can be determined by theta and phi in spherical coordinates. The RCS values can be described as follows[27]:

1 $$ \sigma \,\left( {{\text{dB}}\,{\text{m}}^{2} } \right) \, = {1}0{\text{log}}\left( {\left( {4\pi S/\lambda^{2} } \right)|E_{s} /E_{i} |} \right)^{2} $$

Herein, *S*, *λ*, *E*_*s*_ and *E*_*i*_ represent the area of the target object simulation model, the wavelength of electromagnetic wave, the electric field intensity of scattered wave and the incident wave, respectively.

## Results and Discussion

### Characterization of Shaddock Peel-Derived Carbon Aerogel

As depicted in Fig. 1b, c, the fresh shaddock peel-based precursor shows integrated three-dimensional porous micro-structures without any skeleton fracture, which can be ascribed to the superiority of freeze-drying method. After high temperature pyrolysis, the shaddock peel derived specimen with density of only 0.09 g cm^−3^ can rest quietly on the fragile flower petals (Fig. 1d), indicating the lightweight feature of the as-prepared aerogel. Compared with precursors, the size of 3D porous architecture shrinks to some extent, which can be seen from Fig. 1e–j. Fortunately, the three-dimensional network can still exist even after calcination process, further revealing the advantage of freeze-drying method on the formation of 3D porous network structure. In addition, this interconnected structure can bring abundant electron-transport channels and plenty of pores between nodes and ligaments, which can provide more space for microwave multiple scatter propagation.

**Fig. 2:**
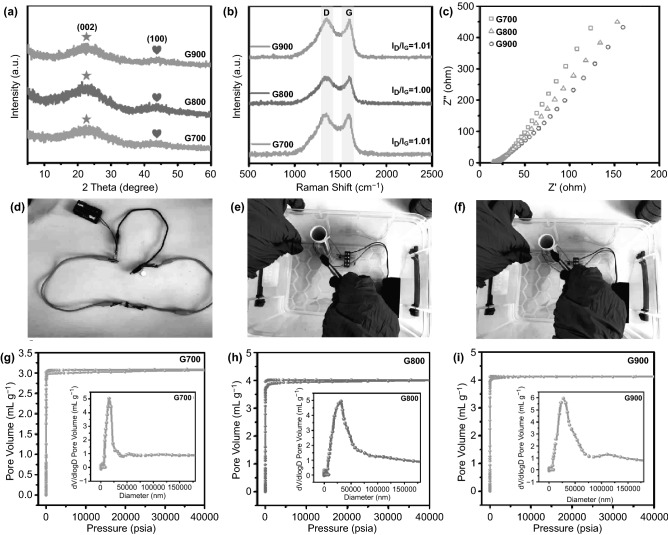
**a** XRD patterns of the obtained samples. **b** Raman spectra of G700/G800/G900. **c** Nyquist plots of the samples. **d**–**f** Digital images of circuit connection and Tesla wireless transmission experiments. **g**–**i** Mercury intrusion and extrusion curves of the as-prepared carbon aerogels with the insert of pore size distributions

XRD patterns of G700/G800/G900 samples are provided in Fig. 2a. Obviously, two broad peaks lie in 24.5° and 43.4° can be well-indexed to the (002) and (100) planes of graphitized carbon. Raman spectra in Fig. 2b further demonstrate the graphitization degree of all shaddock peel-based aerogel products. The prominent peaks at 1350 and 1580 cm^−1^ correspond to D-band and G-band, which are related to disordered carbon and *sp*^2^-bonded carbon, respectively [28]. As for all products, the *I*_D_/*I*_G_ values (intensity ratio of D-band to G-band) are 1.01, 1.00, and 1.01, indicating the same high graphitization degree [29]. That is to say, calcination treatment with different high temperatures has little influence on the bonding state and conductivity of shaddock peel-based carbon aerogel. In order to further investigating electrical conductivity, the electronic impedance of G700/G800/G900 has been measured. As can be seen from the Nyquist plots in Fig. 2c, the size of the semicircles has a descending trend from G700 to G800 and G900, revealing the order of electronic transfer resistance is G700 > G800 > G900 [30]. Namely, the order of electrical conductivity is G900 > G800 > G700. In addition, circuit connection and Tesla wireless transmission experiments demonstrate the steady electrical conductivity of the G800 sample, which are shown in Fig. 2d–f. Obviously, two conductive clips with G800 specimen are directly connected in series to a light-emitting diode (LED) and two small AA-size batteries (Fig. 2d), this LED lamp can keep a stable green brightness. With respect to the Tesla coil (Fig. 2e, f), it is a boosting transformer. By boosting the voltage of the transformer, the primary coil passes a changing current and creates a high voltage in the secondary coil. Essentially, Tesla wireless transmission is a mode of energy transmission via magnetic resonance rather than direct physical contact between the power supply object and electricity demand object [31]. The corresponding principle is that the electrical energy sender and receiver coils constitute a combined magnetic resonance system. When the frequency of the oscillating magnetic field generated by the sending end is consistent with the natural frequency of the receiving end, the receiving end resonates, thus realizing the energy transmission. In a high voltage electric field, neon gas in a neon bubble gives off light by glow discharge. Thus, the neon bulb in Fig. 2e can present an orange light because of strong glow effect. However, if an electric conductor is placed between the power supply and demand objects, the resonance balance will be destroyed and then the electric energy transmission will be blocked. Namely, the neon bulb can not apply glow effect under this circumstance. As depicted in Fig. 2f, due to the excellent electrical conductivity, the shaddock peel derived G800 carbon aerogel can hinder power transfer, which may lead to more dielectric loss. In addition, the unique three-dimensional microcellular structure of the as-prepared carbon aerogels can be further found in Fig. 2g–i. The relevant mercury intrusion and extrusion curves of the as-prepared carbon aerogels exhibit that the total pore volume of G700/G800/G900 samples are 3.07, 4.01, and 4.12 mL g^−1^, respectively. Meanwhile, for G700/G800/G900 specimens, their porosities are 78.70%, 83.53%, and 83.22%, separately, indicating the cellular structure and lightweight feature of the shaddock peel-based carbon aerogels. As illustrated in the pore size distribution curves in Fig. 2g–i, the pore diameters of the as-obtained specimens mainly distribute from nanoscale to microscale, further revealing the 3D porous architecture and lightweight characteristic of the carbon aerogel.

### Thermal Insulation Performance

**Fig. 3:**
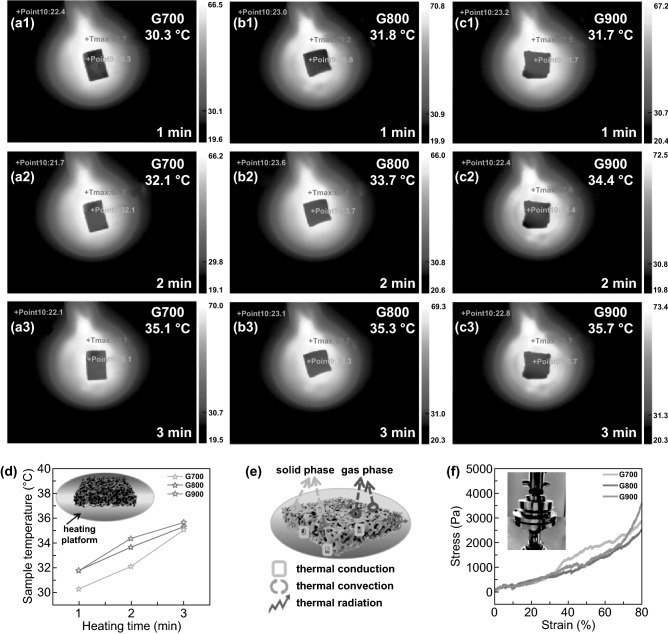
**a**–**c** Thermal infrared images of G700/G800/G900 samples captured at 1/2/3 min, respectively. **d** Heating time versus sample temperature line charts of the biomass-based aerogels. **e** Schematic illustration of the heat transfer mechanism of the 3D porous network. **f** Representative compression stress–strain (*σ*–*ε*) curves of G700/G800/G900 aerogels upon 80% strain

Traditional microwave absorbing powders lack multifunctional features, such as thermal infrared stealth property, heat insulation function, and mechanical performance. Fortunately, due to the superiority of the 3D porous skeleton structure, the shaddock peel-derived carbon aerogels are highly desired for complex application environment. As shown in Fig. 3a–c, taking one piece of each sample and placing them on the platform with the set heating temperature of 70 °C separately. Evidently, the upper surface of all products appears dark, which is similar to the color of surrounding environment. This unique phenomenon reveals the outstanding thermal infrared stealth function of the 3D porous network. The thermal infrared images recorded the temperature variation of the sample top surface for 3 min. The detected temperatures are 30.3, 32.1, and 35.1 °C for G700; 31.8, 33.7, and 35.3 °C for G800; 31.7, 34.4, and 35.7 °C for G900, respectively. As illustrated in Fig. 3d, the slow upward trends directly demonstrate that the shaddock peel-based aerogel possess excellent thermal insulating property. Figure 3e shows the possible heat transfer mechanism of the biomass-based carbon aerogel, including three main types: (1) thermal conduction of solid phase or gas phase, (2) thermal convection of gas in pores and (3) thermal radiation between hole walls and pores [32, 33]. Due to abundant air with lower thermal conductivity take the place of solid phase with higher thermal conductivity, the biomass-based aerogels exhibit superb thermal insulation performance. Satisfactory mechanical property also contributes to wide applications in industrial field [34]. The stress–strain curves have been captured with a strain of 80% at a strain rate of 0.5 mm min^−1^. As depicted in Fig. 3f, the maximum compressive stresses of G700/G800/G900 are 2.815, 2.435, and 3.639 kPa, indicating the favorable mechanical property of the shaddock peel-based aerogels.

### Microwave Absorption Property

**Fig. 4:**
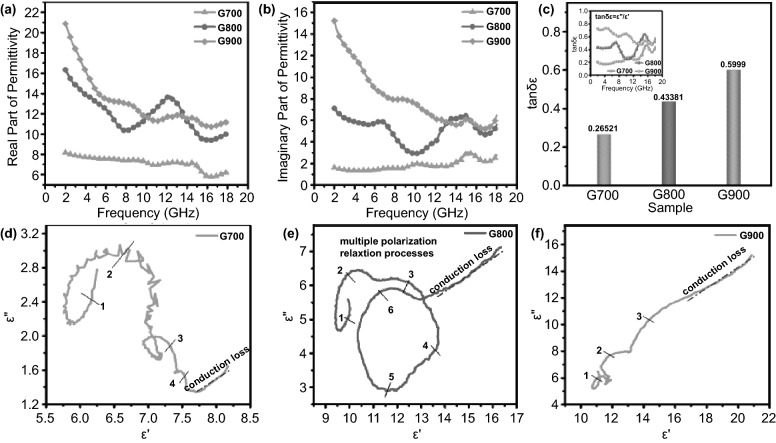
**a**, **b** Frequency-dependent value curves of real part of permittivity (ε′) and imaginary part of permittivity (ε″) of all products. **c** A bar chart of average dielectric loss tangents of each sample. Insert is the line chart of frequency dependent dielectric loss tangents. **d**–**f** Cole–Cole plots of G700, G800, and G900

Taking nonmagnetic ingredient of shaddock peel derived aerogel into account, it can be deduced that dielectric loss plays a leading role in electromagnetic absorbing behaviors. Hence, complex permittivity, including real part (*ε′*) and imaginary part (*ε″*), were measured in the frequency region from 2 to18 GHz. Figure 4a exhibits that the average *ε′* value of G700 is 7.14, the *ε′* value of G800 decreases from 16.35 to 9.94 and the *ε′* value of G900 specimen declines from 20.87 to 11.08. Meanwhile, Fig. 4b displays the average imaginary part permittivity *ε″* values for G700/G800/G900 are 1.85, 5.11, and 7.97, respectively. There is no doubt that the storage (*ε′*) and dissipation (*ε″*) capacity show an upward trend with increasing temperature. Obviously, as the frequency increases, the dielectric response (*ε′* and *ε″* values) gradually decreases for G800 and G900, which can be ascribed to frequency dispersion effect. However, as for G700 sample, the real (*ε′*) and imaginary (*ε″*) permittivity values keep smooth with the same constant, indicating frequency dispersion effect plays a minor role in microwave absorbing performance. In addition, the *ε*_*r*_–*f* curve exhibits several fierce fluctuations in the X and Ku band for G800 and G900 specimens, which may be explained by multiple dipole polarization relaxation behaviors and splendid dielectric attenuation ability of the carbon aerogel [35]. Dielectric loss tangents (tan*δ*_*ε*_ = *ε″*/*ε′*) values of all aerogel samples are 0.26521, 0.43381, and 0.5999, respectively, which can be seen from Fig. 4c [36]. In addition, the top-left inserts signify the frequency dependence of tan*δ*_*ε*_. Based on the above analysis, G900 sample possesses the strongest dielectric dissipation capacity among all samples. According to the Debye theory, the relationship between real part and imaginary part of permittivity can be described as the following equation [37]:

2 $$ (\varepsilon^{\prime } - (\varepsilon_{s} + \varepsilon_{\infty } )/2)^{2} + \, (\varepsilon^{\prime \prime } )^{2} = \, ((\varepsilon_{s} - \varepsilon_{\infty } )/2)^{2} $$

Herein, *ε*_*s*_ signifies the static permittivity and *ε*_*∞*_ represents the relative dielectric permittivity at limiting high frequency. As a rule, if the *ε′*-*ε″* curves present semicircles, which are defined as Cole–Cole semicircles, there will appear Debye relaxation processes [38]. For comparison, the *ε′*-*ε″* curves of shaddock peel-derived carbon aerogel have been drawn in Fig. 4d–f. Obviously, there are 4, 6, and 3 distorted semicircles in the curves of G700/G800/G900 samples, illustrating that G800 specimen experiences more Debye relaxation processes than the other two specimens, which may be caused by graphitized carbon and abundant defects [39]. Due to the existence of three-dimensional interconnected conductive network, conduction loss plays a significant role in dielectric loss. As can be seen from Fig. 4d, the G700 sample presents shorter dash dot line than the other two, manifesting that G800 and G900 samples exhibit more migrating and hopping electrons within the 3D interconnected network. Namely, G800 and G900 samples exhibit enlarged conduction loss ability, which is consistent with increased annealing temperature. Comprehensively considering dual factors of both dipole polarization processes and conduction loss capacity, it can be deduced that G800 specimen shows best microwave absorbing ability.

**Fig. 5:**
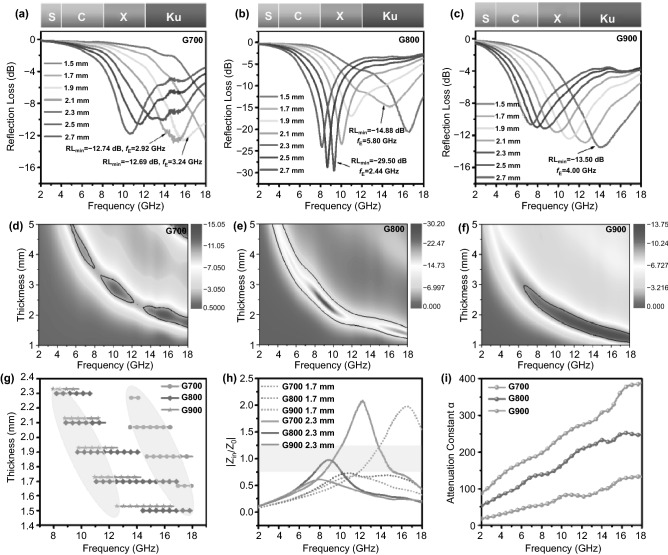
**a**–**c** Reflection loss peaks at relatively thin thicknesses of all shaddock peel-based aerogel samples. **d**–**f** 2D representation of RL values for G700, G800, and G900 samples in the full tested frequency region. **g** Effective bandwidth of all samples. **h** Comparison of impedance matching |*Z*_*in*_/*Z*_*0*_| values at 1.7 mm and 2.3 mm of G700, G800, and G900. **i** Attenuation constants of G700/G800/G900

It is indispensible to verify the aforementioned speculation is correct or not. Thus, on the basis of transmission line theory, the reflection loss (*RL*) values of as-prepared samples have been evaluated to intuitively examine and weigh microwave absorbing performance. Within the whole testing frequency range, *RL* peaks at relatively thin thicknesses and 2D representation of RL values for all shaddock peel-based aerogel samples are summarized in Fig. 5a–f. As is known to all, RL values can be obtained using the following formulas [40–42]:

3 $$ {\text{RL}} = 20\log \left| {\left( {Z_{{{\text{in}}}} - Z_{0} } \right)/(Z_{{{\text{in}}}} + Z_{0} )} \right| $$

4 $$ Z_{{{\text{in}}}} = \, Z_{0} \left( {\mu_{r} /\varepsilon_{r} } \right)^{1/2} \tanh \left[ {j\left( {2\pi fd/c} \right)\left( {\mu_{r} \varepsilon_{r} } \right)^{1/2} } \right] $$

Herein, *Z*_*in*_ represents the input impedance value of the microwave absorbing materials and *Z*_*0*_ signifies the impedance value of air. Besides, *μ*_*r*_ stands for complex permeability, *ε*_*r*_ is complex permittivity, *f* represents the full measuring frequency, *d* means the thickness of absorber, and *c* signifies the velocity of light, respectively. Thanks to the efficient microwave absorbing performance (*RL* < − 10 dB, 90% microwave can be effectively absorbed), all of the 3D porous aerogel samples in this work can be used in practical applications at proper thicknesses. With the thickness increasing from 1.5 to 2.7 mm (Fig. 5a–c), the reflection loss peaks of the as-obtained samples gradually shift to low frequency region. This phenomenon may be related to the quarter wavelength theory [43]: *f*_*m*_ = *nc*/*4t*_*m*_(*ε*_*r*_*μ*_r_)^1/2^, where *t*_*m*_ represents the thickness and *f*_*m*_ is the peak frequency. With regard to specific microwave absorbing properties, G700 sample shows the minimum reflection loss value of only − 12.74 dB and a narrow bandwidth of only 3.24 GHz, which is unsatisfied. As for G800, the optimum reflection loss value (*RL*_min_) attains − 29.50 dB at the thickness of merely 2.3 mm in X band, and the effective frequency range (*f*_*E*_) reaches 5.80 GHz (from 11.08 to 16.88 GHz) at a relatively thin thickness of 1.7 mm in Ku band. Unfortunately, G900 specimen exhibits *RL*_min_ of only − 13.50 dB with an effective bandwidth of 4.00 GHz at high frequency. Additionally, Fig. 5d–f presents the 2D color fill contour plots of reflection loss values. It should be noted that G800 sample can acquire the maximum area marked out by black bold lines, where the RL values are less than − 10 dB. Besides, Fig. 5g provides direct comparison of the bandwidth information for all products, suggesting that G800 owns the widest bandwidth at thin thicknesses among all shaddock peel-based aerogel samples. Therefore, in this case, G800 sample possesses optimum microwave-absorbing performance, which can be attributed to favorable impedance matching property and extremely high attenuation ability [44]. Speaking of impedance matching, it can be given on the basis of the following equations [45]:

5 $$ Z = \, \left| {Z_{{{\text{in}}}} /Z_{0} } \right| $$

6 $$ Z_{{{\text{in}}}} = \left( {\mu_{r} /\varepsilon_{r} } \right)^{1/2} Z_{0} $$

It is generally accepted that if there exhibit almost none reflection between air and the upper surface of the microwave absorbing materials, the value of impedance matching (*Z*) should be close to 1 [46]. If so, it will help electromagnetic wave enter into the internal of the as-obtained material as much as possible. When the thickness is 1.7 and 2.3 mm, *Z* values of 0.75–1.25 occupy the frequency range from 7.52 GHz to 18 GHz (Fig. 5h). Herein, the order of suitable impedance matching performance should be G700 > G800 > G900. Apart from the above factor, the integral attenuation capacity of the microwave absorber (attenuation constant *α*) can be expressed as [47]:

7 $$ \alpha = \frac{\sqrt 2 \pi f}{c} \times \sqrt {(\mu^{{\prime \prime }} \varepsilon^{{\prime \prime }} - \mu^{{\prime }} \varepsilon^{{\prime }} ) + \sqrt {(\mu^{{\prime \prime }} \varepsilon^{{\prime \prime }} - \mu^{{\prime }} \varepsilon^{{\prime }} )^{2} + (\mu^{{\prime }} \varepsilon^{{\prime \prime }} + \mu^{{\prime \prime }} \varepsilon^{{\prime }} )^{2} } } $$

As shown in Fig. 5i, the order of splendid attenuation characteristic should be G900 > G800 > G700. To sum up, both the synergistic effect of suitable impedance matching characteristic and outstanding attenuation property contribute to microwave absorbing performance, explaining why G800 shows optimum electromagnetic absorption among all aerogel samples.

**Fig. 6:**
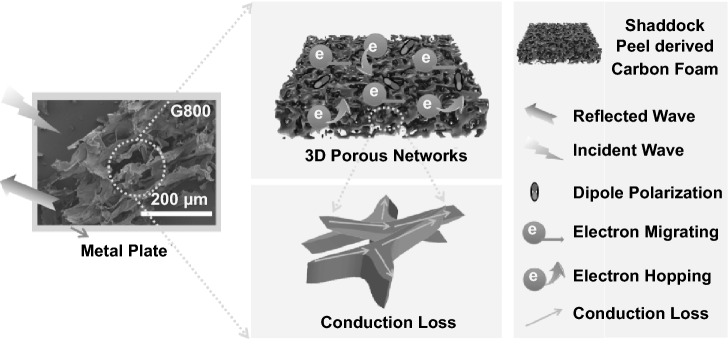
Schematic illustration of microwave absorption mechanisms for 3D shaddock peel-derived carbon aerogel

Based on the metal back model, a possible microwave absorption mechanism of the 3D shaddock peel-derived carbon aerogel has been given in Fig. 6. Thanks to the interlinked network structure of the as-prepared porous carbon aerogel, several points can be beneficial for improving microwave absorption performance. First of all, strong dipole polarization mechanisms and relaxation mechanisms, induced by the incompatibility of dipoles migrating and external electric field, are useful for enhancing dielectric loss ability [48]. Moreover, the aforementioned Cole–Cole curves of the as-synthesized 3D carbon aerogels (Fig. 4d–f) can confirm the existence of dipole polarization process and Debye relaxation process. Besides, the conductive routes of the biomass-based carbon network can bring active migrating electrons and hopping electrons, which can be available for conduction loss property [49]. Owing to the unique three-dimensional skeleton architectures with high porosity, multiple scattering propagation paths of microwave can be obtained by the shaddock peel-derived carbon aerogel [50]. In addition, sufficient internal absorbing behaviors can increase the exhaustion of microwave energy in the carbon aerogel. Furthermore, proper impedance matching has a significant effect on absorption efficiency of electromagnetic wave.

### RCS Simulation Results

**Fig. 7:**
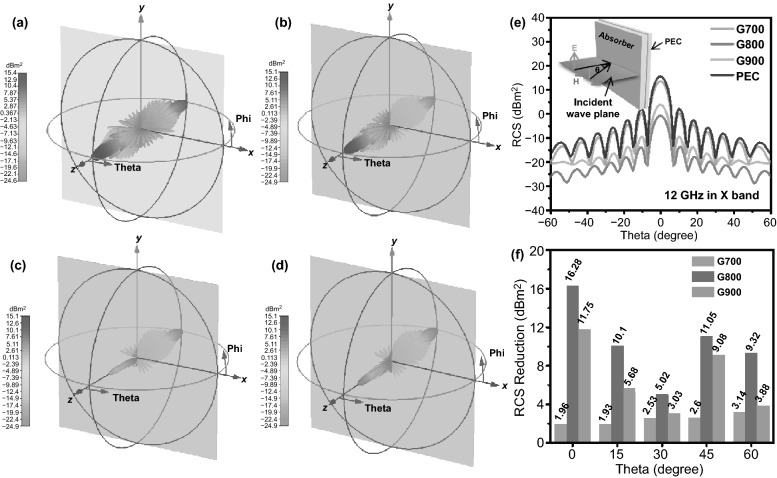
CST simulation results of the samples: **a** perfect conductive layer (PEC), **b**–**d** the perfect conductive layer covered with G700, G800, and G900 samples. **e** RCS simulated curves of PEC and all shaddock peel-derived carbon aerogel products under different scanning angles. **f** Comparison of RCS reduction values of G700/G800/G900 samples

CST simulation results of the perfect conductive layer (PEC) and the PEC layer covered by G700, G800, and G900 samples are shown in Fig. 7a–d, which can reflect the real far field condition of microwave absorbing performance of the as-prepared aerogels. In this simulation model, the positive *Z* axis is selected to be the direction of incidence and theta is defined as the detection angle. With the angle variation range from − 60° to 60° at 12 GHz, the pristine PEC layer and all as-synthesized samples display three-dimensional radar wave scattering signals of different intensities. Evidently, PEC plate exhibits the maximum scattering signal, which can be observed from Fig. 7a. The simulated appearance result of the square flat plate covered by G800 sample is much less than PEC and other objects, manifesting the minimum radar cross-sectional (RCS) value of G800 sample. As a validation, 2D curves of RCS values have been depicted in Fig. 7e. When microwave incident perpendicularly to the model plane, the reflected electromagnetic wave can occupy a larger proportion, which can be seen from Fig. 7e. With the deviation of detection angle, the RCS values gradually decrease from 0° to ± 60^o^ with several fluctuations. As compared to PEC, G700 and G900, there is no doubt that G800 exhibits the lowest RCS values over the angle range of − 60° to 60° at 12 GHz in X band. The RCS values of G800 specimen are less than − 10 dB m^2^ over the range of − 60° < theta < − 6° and 6° < theta < 60° at the coating thickness of 1.7 mm. This simulated result corresponds well with the outstanding microwave absorbing property in Fig. 5. In order to further testifying the above description, the relevant bar charts of comparing the RCS reduction values (the RCS values of PEC minus that of samples) are presented in Fig. 7f. When theta arrives at 0°, the maximum RCS reduction values can reach 16.28 dB m^2^ for G800 sample. That is to say, the obtained aerogel possesses fabulous radar wave attenuation property, which can suppress the scattering and reflecting electromagnetic waves from the surface of PEC. Therefore, this kind of shaddock peel-based carbon aerogel can be appropriate for practical applications.

## Conclusion

In conclusion, a series of shaddock peel-based carbon aerogels were prepared via a facile freeze-drying method and a subsequent high temperature treatment. Thanks to the interlinked conductive network with plenty of pore structures, the as-synthesized specimens show multi-functions as the following. Firstly, wonderful thermal infrared stealth property and heat insulation performance can not only protect the object from detection but also ensure availability of devices in high temperature environment. Then, promising mechanical performance allows equipments to reuse in daily routine. Except that, superb electrical conductivity of the as-obtained aerogels can be helpful to efficient dielectric loss ability, which may be beneficial for microwave absorbing performance. In detail, the obtained sample reaches an effective bandwidth (the *RL* values below − 10 dB) of 5.80 GHz in X and Ku band at a relatively thin thickness of 1.7 mm. Also, this work provides CST simulation data for the biomass-based carbon aerogels, which can be an intelligent design strategy for microwave absorption. Since the G800 sample covered model possesses smaller RCS values than PEC model, demonstrating the splendid microwave absorption performance. Therefore, this work provides lightweight shaddock peel derived microwave absorbing aerogels with multiple functions and brings novel RCS simulation method for predicting microwave absorption in practical application situation, which can of great significance for many fields in the future.
